# Amino acid infusion during anesthesia attenuates the surgery induced decline in IGF-1 and diminishes the "diabetes of injury"

**DOI:** 10.1186/1743-7075-4-2

**Published:** 2007-01-09

**Authors:** Mats KEB Wallin, Eva Selldén, Staffan Eksborg, Kerstin Brismar

**Affiliations:** 1 Department of Anesthesiology and Intensive Care, Karolinska University Hospital, Stockholm, Sweden; 2 Department of Woman and Child Health Karolinska Institutet, Stockholm, Sweden; 3 Department of Molecular Medicine and Surgery, Karolinska Institutet, Stockholm, Sweden

## Abstract

**Background:**

Surgery, commonly performed after an overnight fast, causes a postoperative decline in the anabolic and glucose lowering insulin-like growth factor-1 (IGF-1). Clinical fasting studies have exhibited a positive correlation between IGF-1 and nitrogen balance during different conditions. A perioperative amino acid infusion changes nitrogen balance and might thereby influence serum IGF-1. We hypothesized that amino acid infusion would enhance IGF-1 and thereby might influence glucose homeostasis after surgery. In this study we examined two different regimes of perioperative amino acids infusion.

**Methods:**

24 females scheduled for abdominal hysterectomy were randomized into three groups; Ringer's solution infusion throughout anesthesia (Group B), amino acid infusion throughout anesthesia (Group C) and amino acid infusion 1 hour before anesthesia and during 1.5 hrs of surgery (Group D). Six female volunteers, who were not operated, but received the same amino acids infusion after fasting, served as controls (Group A). Fasting levels of IGF-1, Insulin-like growth factor binding protein-1 (IGFBP-1), insulin and P-glucose were studied prior to, and four days following, operation. Homeostasis model assessment (HOMA) was used as an index of insulin resistance. Non-parametric statistical methods were used.

**Results:**

During the study the Ringer-group exhibited a decrease in IGF-1 and an increase in insulin and plasma glucose after surgery. Within the other groups there were no significant alterations over time after surgery, with the exception of a postoperative decrease in IGF-1 in group D. Group C had higher IGF-1 levels compared to group B on all days. Also, group D had higher IGF-1 levels than group B on day 2 – 4. From baseline to the first postoperative day there was a significant increase in HOMA and IGFBP-1 in groups B and C. These changes were not found in group D, in which insulin, glucose, HOMA and IGFBP-1 did not change. Amino acid infusion to the volunteers did not affect any of the variables studied.

**Conclusion:**

Amino acid infusion during surgery attenuates the decrease in IGF-1 and diminishes the "diabetes of injury".

## Background

Elective surgery is traditionally performed after an overnight fast in order to reduce the risk of aspiration. It has recently been shown that preoperative carbohydrate treatment reduces insulin resistance after surgery with a shorter length of hospital stay [1]. Shorter hospitalization has also been reported after amino acid treatment during surgery [2]. The insulin-like growth factor-1 (IGF-1) is a common denominator for amino acid and carbohydrate metabolism [3]. The anabolic axis of growth hormone (GH) and IGF-1 is sensitive to nutritional variations [4,5]. Fasting and catabolic states cause an uncoupling between IGF-1 and GH with high serum levels of GH and low IGF-1 [6]. Both sufficient energy and protein intake are necessary for a normal IGF-1 response to GH. IGF-1 stimulates amino acid and glucose uptake as well as protein synthesis in muscle [3,7]. In humans, IGF-1 also reduces B-glucose in parallel with insulin [8-10]. Circulating IGF-1 is produced in the liver and more than 99 % is bound to binding proteins (IGFBPs, 1–6) [4,11]. About 80–90% is bound to a stable ternary protein complex consisting of IGF-1, IGFBP-3 and Acid Labile Subunit (ALS) with a long half-life (≈15 h)[12,13]. The ternary complex stabilize the concentration of IGF-1 in plasma and regulates the efflux of free IGF-1 from the vascular space [14]. The smaller IGFBPs (IGFBP-1, -2, -4, -6) form binary complexes with IGF-1 and do not bind to ALS. Only the free fraction, which is less than 1% of total IGF-1, is supposed to be active at the receptor[11]. After surgery [15,16] and during pregnancy [17] and critical illness [18,19] an proteolytic activity is observed which reduces the half-life of IGF-1. In addition, serum levels of the smaller binding proteins are often elevated during critical illness [20].

Insulin-like growth factor binding protein-1 (IGFBP-1), produced in the liver [11,21], is an important regulator of free IGF-1. Its production is inhibited by insulin at the transcription level [22] and is stimulated by glucagon [23], epinephrine [24] and cytokines [25]. IGFBP-1, free IGF-1 and insulin all of them have a half-life about 5–10 minutes [11,13,26].

IGF-1 is also produced in other tissues, such as muscle, and acts as a paracrine and autocrine growth factor [27]. This local production is also dependent on GH, and nutrition [4,28].

A number studies in fasting subjects have described a close correlation between total IGF-1 and nitrogen balance [4,28]. Earlier studies have reported increased renal utilization of amino acids with increasing blood concentration [29]. Thus infusion of amino acids ought to be utilized by patients during surgery. In humans amino acids act both as substrates and transmitters. The effect of an amino acid infusion during surgery might thus be different if it is given before or after induction of anesthesia. The postoperative effect of amino acid infusion given during anesthesia and surgery concerning IGF-1 and glucose homeostasis has previously not been elucidated. The aim of this pilot study was to investigate the effect of two regimes of amino acids infusion, before and during hysterectomy, on the surgery induced decrease in IGF-1 and "diabetes of injury" [30].

## Methods

Thirty women were included in the study. Patients with known metabolic, hepatic or renal disorders were not included in this study and none of the studied subjects had reported any weight changes prior the investigation. Neither of them was on any drug treatment. They were all informed of the study and its risks before they consented to participate. The study protocol was approved by the Ethics Committee at Karolinska Hospital.

### Study groups

The subjects were divided into four study groups. Six female volunteers acted as a control group from the normal population (group A). They were fasting over night but otherwise had no food restrictions during the study and were given an i.v. amino acid infusion for 2.5 h, in an amount that was similar to the two amino acid treated patient groups. The volunteers were not hospitalized or subjected to anesthesia or surgery. Twenty-four female patients scheduled for hysterectomy due to myoma were randomly divided into three groups (groups B, C, D). Eight control patients were anaesthetized and received no amino acids, but a corresponding volume (126 mL/h) of Ringer's solution, (group B). Another group of 8 patients received amino acid infusions starting at induction of anesthesia and continuing throughout surgery until awakening (group C). In group D, eight patients were given amino acids during 1 h before induction of anesthesia and for additional 1.5 h into anesthesia and surgery.

### Anesthesia

Prior to anesthesia and surgery all patients were on an unrestricted mixed diet. The estimated intake of nitrogen and energy 24 h before the study were of the same magnitude in all groups. They were all fasted over-night and got an oral premedication of lorazepam 1 mg 1 h prior to anesthesia. Before induction of anesthesia two cubital veins were catheterized. One catheter for amino acid infusion or acetated Ringer's solution was inserted and advanced centrally 30 cm to reach a tip position in the subclavian vein. The venous catheter in the other arm was used for anesthetic drug administration. Anesthesia was induced with thiopental, 5 mg/kg, and the trachea was intubated after a bolus dose of atracurium 0.5 mg/kg followed by a continuous infusion of 0.5 mg/kg/h. Anesthesia was maintained with 1–2% of isoflurane in O_2_/N_2_O. Fresh gas flows were set at 2 L/min of O_2 _and 4 L/min of N_2_O, using a partial rebreathing circuit (Dameca ventilator 109 40, Dameca, Rødøvre, Denmark). Usual monitors were used. Before the start of surgery fentanyl 3 μg/kg was given. The atracurium infusion was stopped 0.5 h before the end of the operation, and muscle relaxation was antagonized with 2.5 mg of neostigmine and 0.5 mg of glycopyrrolate at the end of surgery. Postoperatively, pain relief was standardized and given according to routine as needed.

During anesthesia and surgery, 500 mL/h of Ringer's solution was infused i.v. in all patients. After emergence from anesthesia, 1000–1500 mL of a 5 % glucose solution was administered in all patients until the next morning. The first postoperative day the patients were on a liquid diet. From the second day after surgery the patients had regular meals. All postoperative energy and nitrogen intake was daily recorded throughout the study. Energy and nitrogen intake were equal during each postoperative day in the three patient groups.

### Amino acid infusion

All studies commenced at 7.30 a.m Healthy volunteers (group A) reported in the morning after an overnight fast. They received a balanced amino acid mixture (Vamin^® ^18 g N/L, Pharmacia, Stockholm, Sweden) at a rate of 126 mL/h, corresponding to 240 kJ of energy per h. The mixture of amino acids in Vamin^® ^18 g N/L is presented in Table 1. Thus, during 2.5 h of infusion, the subjects in group A received 600 kJ of extra energy from amino acids and totally 397 mmoles nitrogen was infused. The patients in group D, in which the amino acid infusion started 1 h before induction of anesthesia and continued during the first 1.5 h of anesthesia and surgery, received exactly the same amounts of energy and nitrogen, *i.e*. 600 kJ and 397 mmoles, respectively. The patients in group C received an identical amino acid infusion throughout anesthesia and obtained 384 ± 24 mmoles (mean ± SEM) of nitrogen. The patients' ward nurses, as well as surgeons, were unaware of whether amino acids or saline was given. Otherwise the study was not blinded.

**Table 1:** The content of amino acids in – Vamin^® ^18 – per 1000 mL.

glycine 7.9 g	histidine 6.8 g	proline 6.8 g
aspartate 3.4 g	isoleucine 5.6 g	serine 4.5 g
glutamate 5.6 g	leucine 7.9 g	threonine 5.6 g
alanine 16.0 g	lysine 9.0 g	tryptophan 1.9 g
arginine 11.3 g	methionine 5.6 g	tyrosine 230 mg
cysteine 560 mg	phenylalanine 7.9 g	valine 7.3 g.

### Measurements and analyses

Blood samples were taken, after an overnight fast, in the mornings of the day of surgery and during the first four postoperative days. Blood samples were stored cold and centrifuged within 2 h. Serum was frozen at -80°C. Samples were analyzed at the same occasion after the study was completed. IGF-1, IGFBP-1, insulin and plasma-glucose were analyzed. Serum concentrations of total IGF-I were determined by RIA after acid ethanol extraction to minimize interference with IGFBPs [31]. The truncated des (1–3) IGF-I that has reduced affinity for IGFBPs was used as the radioligand in the IGF-I RIA. IGFBP-1 was determined in serum by RIA as described by Povoa et al. [32].

Insulin was measured with a commercial RIA-kit, Pharmacia Insulin (Pharmacia, Stockholm, Sweden) and plasma glucose was analyzed by an enzymatic electrochemical method using a glucose analyzer (YSI, Yellow Springs, OH).

HOMA (Homeostasis model assessment) a index of insulin resistance: p-glucose × p-insulin/22.5 [33].

### Statistics

Non-parametric statistical methods were used for the statistical analyses since the biochemical data was not normally distributed. Mann-Whitney test was initially used for preoperative comparisons between groups. Friedmans ANOVA was used to test postoperative alterations of the variables in the groups. If the ANOVA showed significance another Mann-Whitney test was performed for each postoperative day for comparisons between groups after surgery.

Finally, Wilcoxon signed rank test was done for a comparison of preoperative baseline levels with the values the day after surgery. All statistical analyses were performed with SPSS (Chicago, Il). A p value ≤ 0.05 was considered as an indication of statistical significance.

## Results

There were no significant differences between the four groups with regard to age, weight, and height (Table 2). Duration of anesthesia and surgery were also similar in the three operated patient groups (Table 2).

**Table 2:** Demographic data, duration of anesthesia and surgery for 24 patients and 6 volunteers without surgery.

Study Groups	n	Age (year)	Weight (kg)	Height (cm)	Duration of anesthesia (min)	Duration of surgery (min)
Group A	6	50 ± 3	63 ± 3	170 ± 2	-	-
Group B	8	50 ± 2	64 ± 2	165 ± 1	125 ± 8	92 ± 6
Group C	8	48 ± 1	68 ± 3	163 ± 2	121 ± 9	94 ± 9
Group D	8	48 ± 2	61 ± 1	164 ± 2	120 ± 5	87 ± 4

Demographic data, duration of anesthesia and surgery for 24 patients scheduled for hysterectomy and 6 volunteers without surgery. Group A = Amino acid infusion but no anesthesia or surgery; Group B = Ringer's solution infusion throughout anesthesia; Group C = Amino acid infusion throughout anesthesia; Group D = Amino acid infusion started 1 h before anesthesia and during 1.5 h of surgery. Values are mean ± SEM.

**Figure 1:**
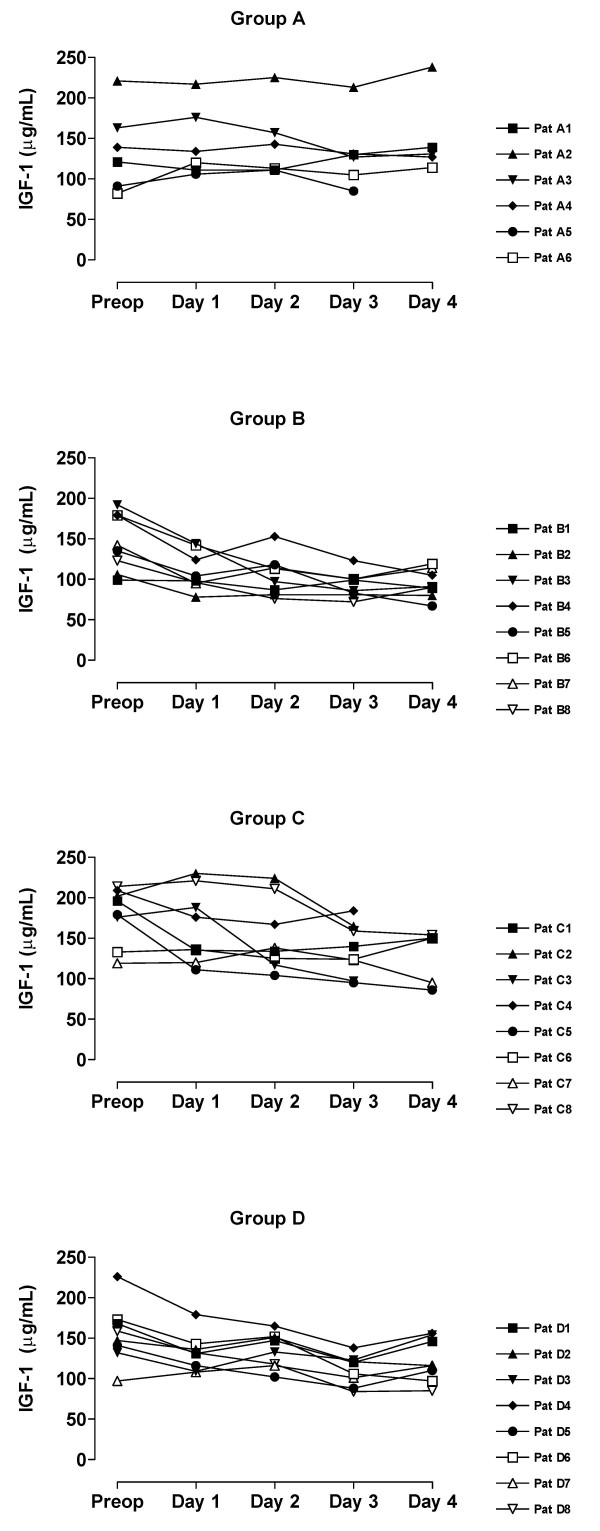
Fasting IGF-1 for each patient sorted by groups.

**Figure 2:**
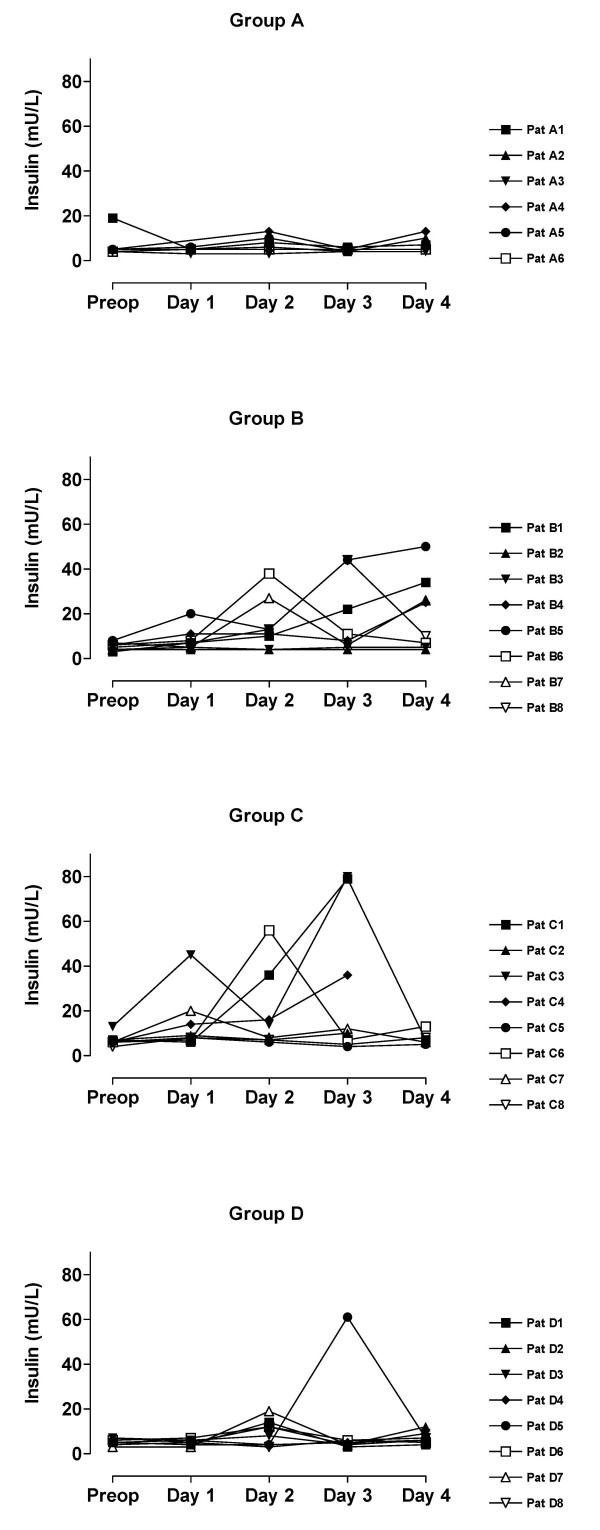
**Fasting insulin for each patient by sorted groups**. Patient D5:s sample day 3 is probably not a true fasting value since it is only at this day she has increased levels of insulin and glucose.

**Figure 3:**
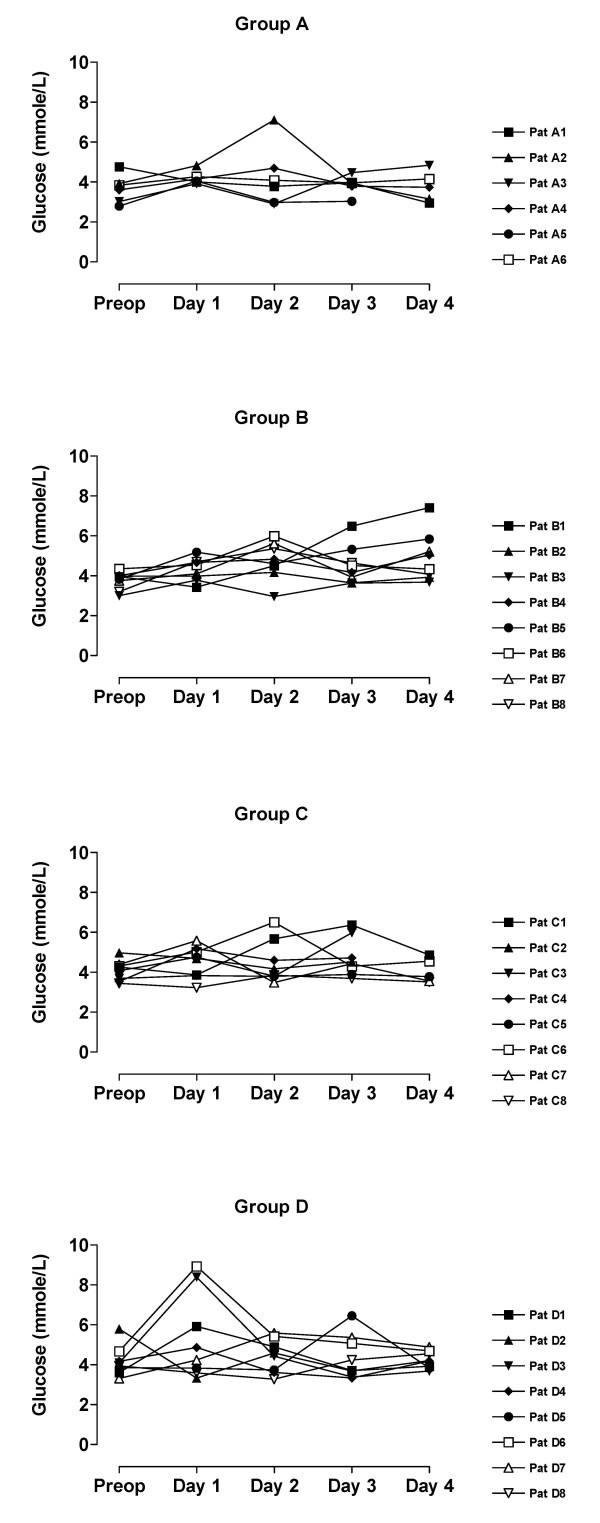
**Fasting P-glucose for each patient sorted by groups**. Patient D5:s sample day 3 is probably not a true fasting value since it is only at this day she has increased levels of insulin and glucose.

**Figure 4:**
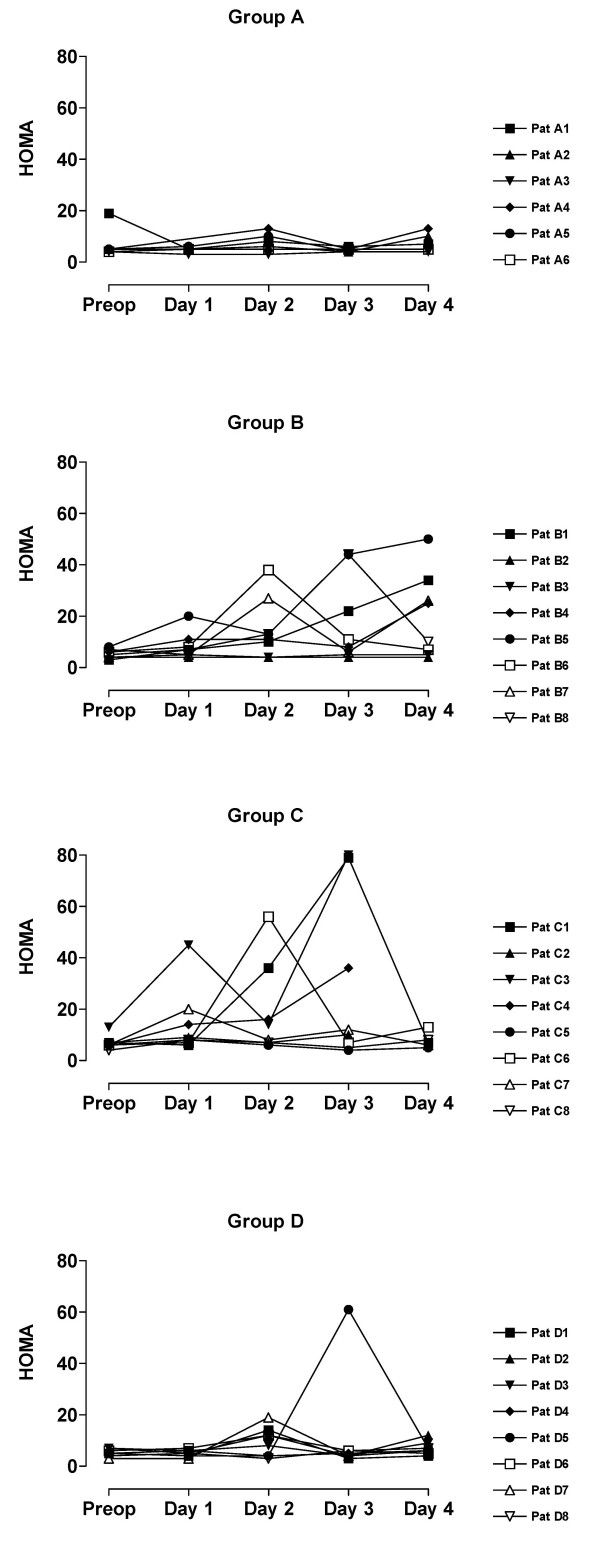
**HOMA for each patient sorted by groups**. Patient D5:s sample day 3 is probably not a true fasting value since it is only at this day she has increased levels of insulin and glucose.

**Figure 5:**
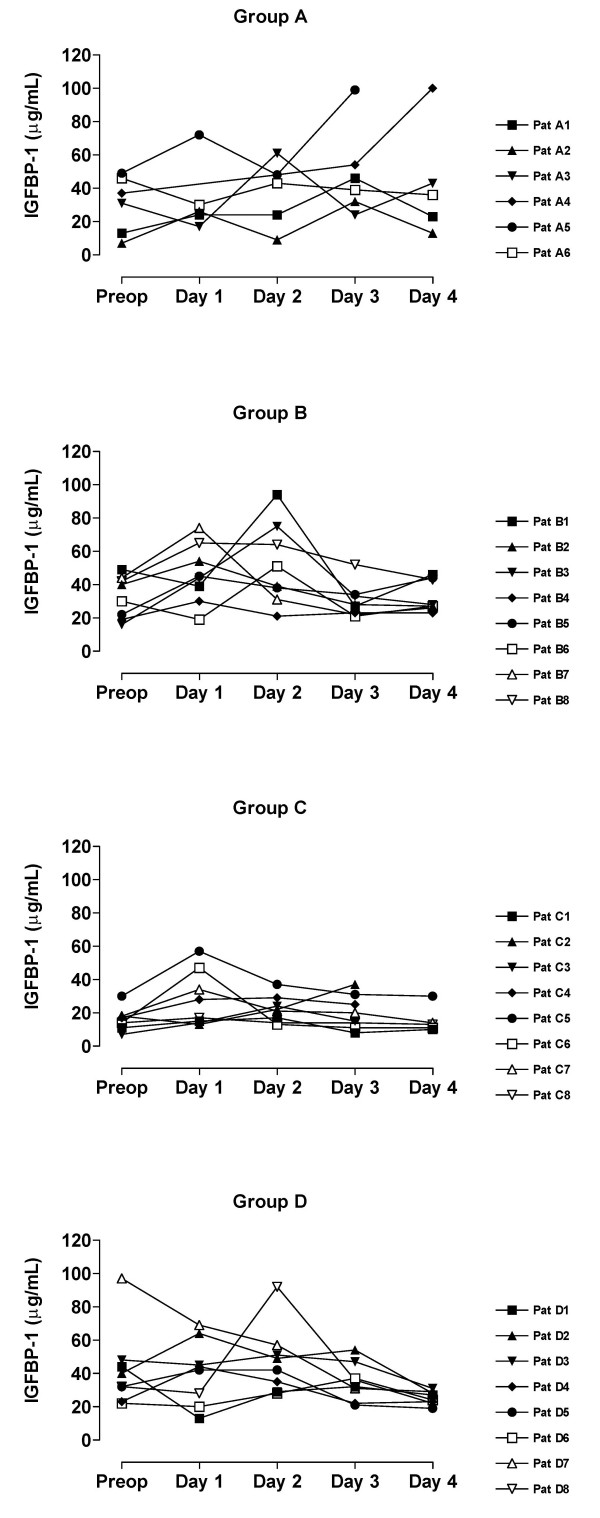
Fasting IGFBP-1 for each patient sorted by groups.

**Figure 6:**
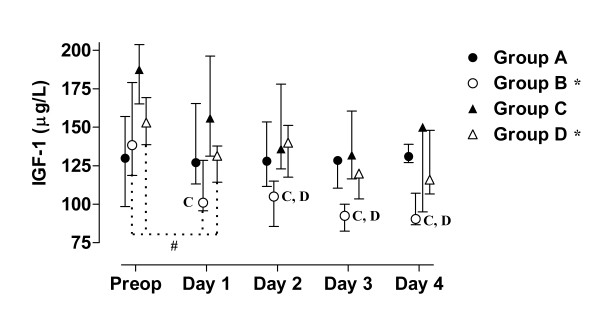
**Median, first and third quartile for IGF-1 by groups**. Patient D5s sample day 3 is withdrawn in these figure since it is considered to be a false fasting sample (figure 2 - 4). Significant alteration (p < 0.05) tested by Friedman ANOVA during the study is marked *. Postoperative significant (p < 0.05) differences in IGF-1 between group B and the amino acid groups are marked C and D respectively. Significant (p < 0.05) difference between baseline and the first postoperative day is marked #.

**Figure 7:**
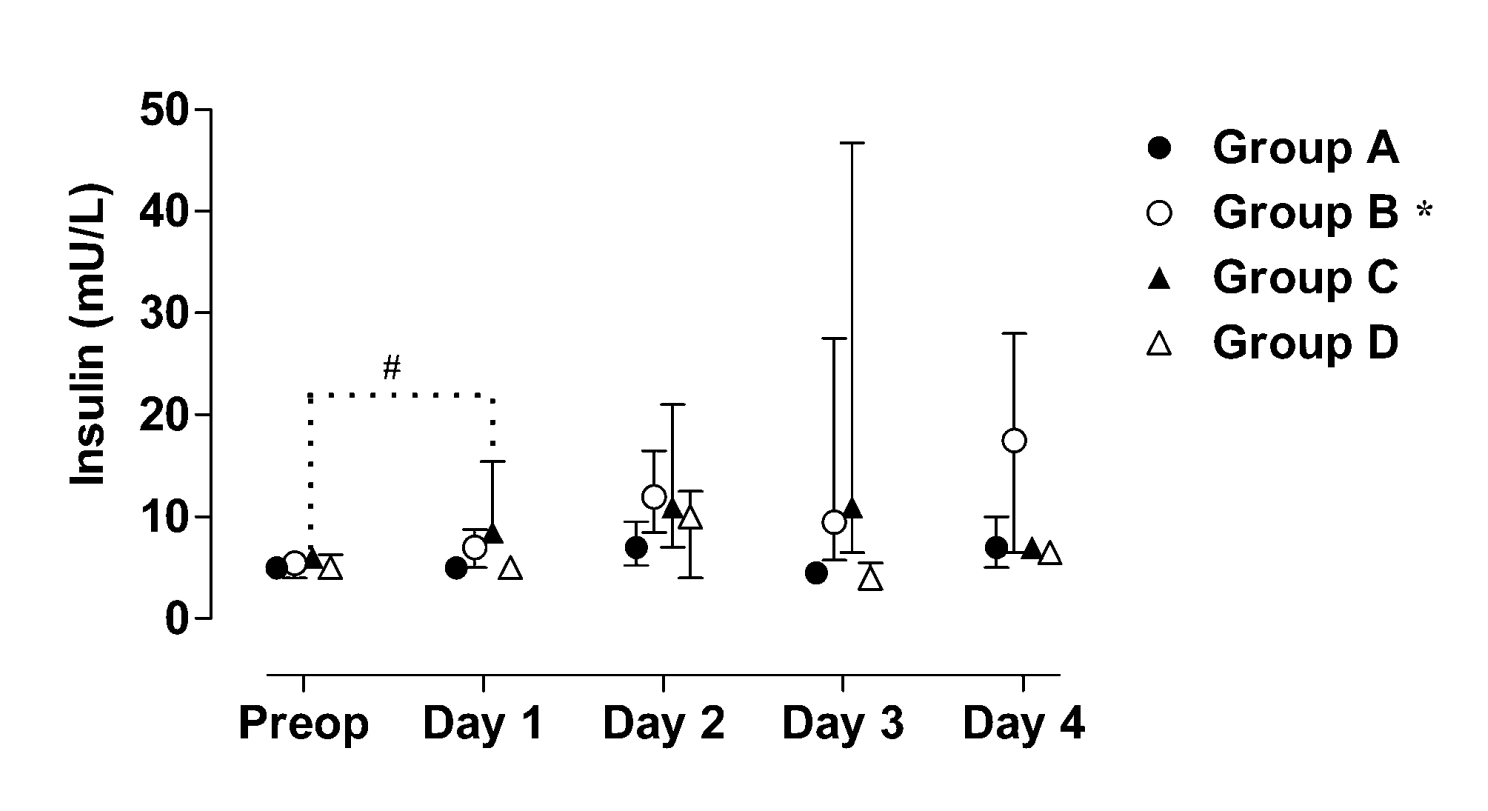
**Median, first and third quartile for insulin by groups**. Patient D5s sample day 3 is withdrawn in these figure since it is considered to be a false fasting sample (figure 2 - 4). Significant alteration (p < 0.05) tested by Friedman ANOVA during the study is marked *. Significant (p < 0.05) difference between baseline and the first postoperative day is marked #.

**Figure 8:**
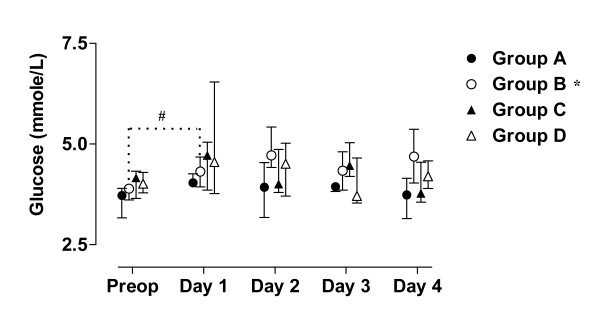
**Median, first and third quartile for P-glucose by groups**. Patient D5s sample day 3 is withdrawn in these figure since it is considered to be a false fasting sample (figure 2 - 4). Significant alteration (p < 0.05) tested by Friedman ANOVA during the study is marked *. Significant (p < 0.05) difference between baseline and the first postoperative day is marked #.

**Figure 9:**
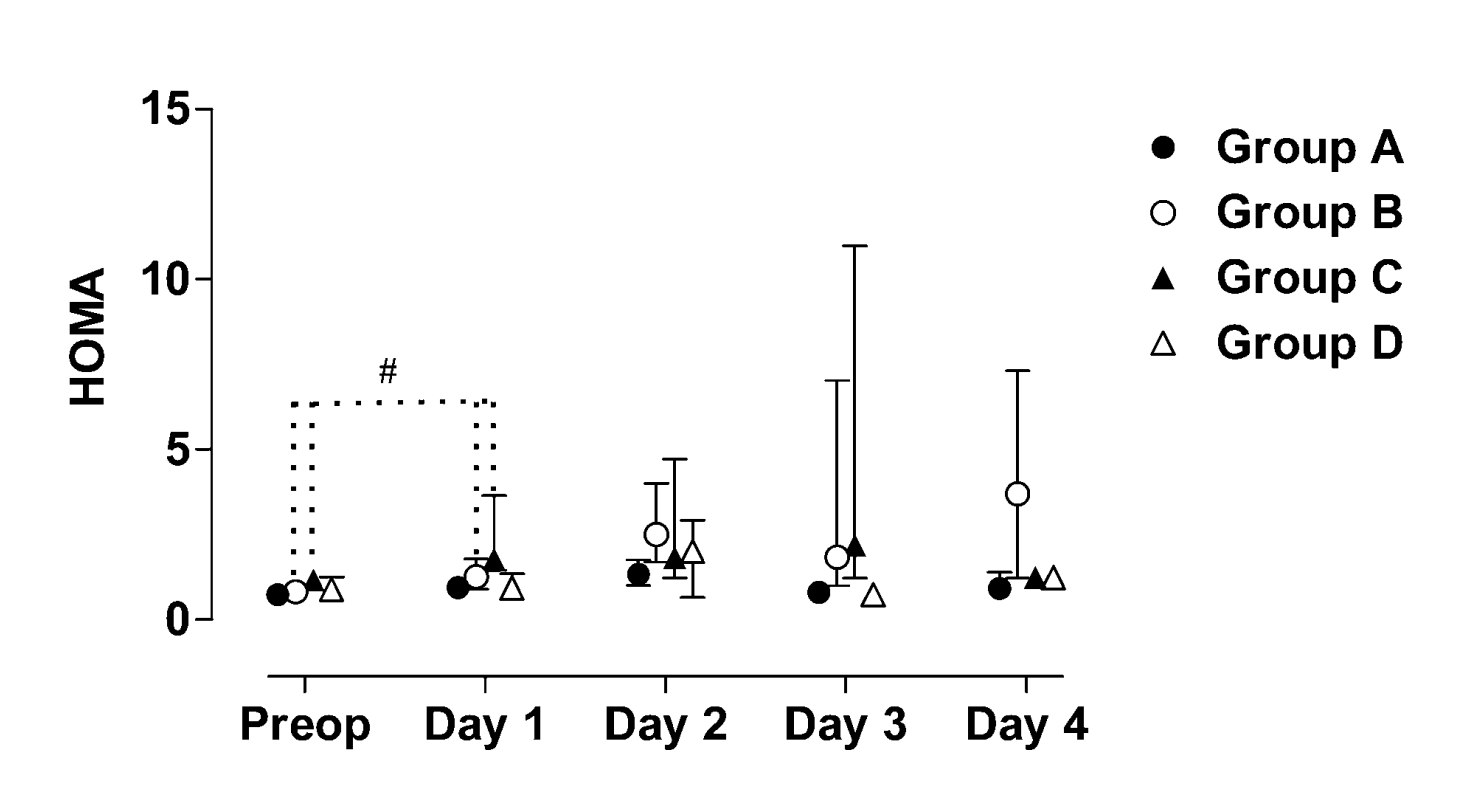
**Median, first and third quartile for HOMA by groups**. Patient D5s sample day 3 is withdrawn in these figure since it is considered to be a false fasting sample (figure 2 - 4). Significant (p < 0.05) difference between baseline and the first postoperative day is marked #.

**Figure 10:**
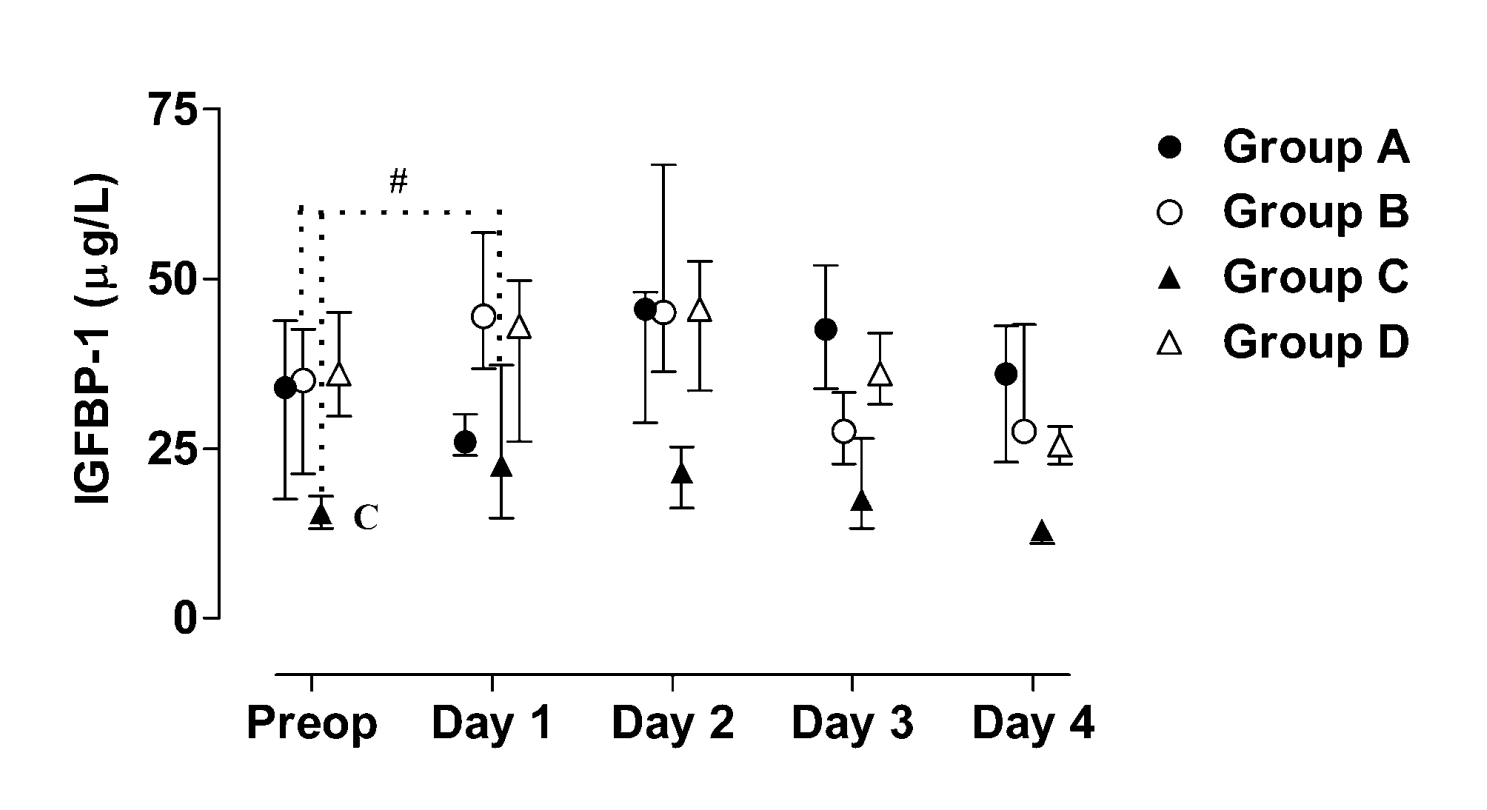
**Median, first and third quartile for IGFBP-1 by groups**. Patient D5s sample day 3 is withdrawn in these figure since it is considered to be a false fasting sample (figure 2 - 4). Preoperative difference between group C and the other groups in IGFBP-1 is marked C. Significant (p < 0.05) difference between baseline and the first postoperative day is marked #.

The value of blood analyses are displayed by groups for each single patient and variable in Figure 1, 2, 3, 4, 5. Results are also presented in Figure 6, 7, 8, 9, 10 as median, first and third quartile for each analyte and is also described in the text below. Among the operated patients, group C had a significantly lower level of IGFBP-1 before surgery than group B and D (Figure 5 &10). Regarding the other variables, there were no significant differences between the groups at baseline.

During the study period Group B, the Ringer-group, exhibited a decrease in IGF-1 (from 139 to 91 μg/L; p = 0.002, Figure 1 &6) and an increase in insulin (from 6 to18 mU/L; p < 0.02, Figure 2 &7) as well plasma glucose (from 3.9 to 4.7 mmol/L; p < 0.05, Figure 3 &8) after surgery. HOMA was increased (from 0.8 to 3.7) but did not reach significance (p = 0.07, Figure 4 &9). IGFBP-1 was unchanged (Figure 5 &10). Within the other groups there were no significant alterations over time after surgery in any variable, with the exception of IGF-1 in group D which decreased postoperatively from 153 to116 μg/L (p < 0.05, Figure 1 &6). Group C had significantly higher IGF-1 levels compared to group B on all postoperative days (p < 0.05, Figure 6). Also group D had significantly higher IGF-1 levels than group B, but only on day 2 – 4 after surgery (p < 0.05, Figure 6). No significant differences in IGF-1 levels occurred between groups C and D.

From baseline to the first postoperative day there was a significant increase in group B and C in HOMA (from 0.8 to1.3 and 1.2 to1.8 respectively; p < 0.05, Figure 9) and IGFBP-1 (from 35 to45 μg/L and 16 to23 μg/L; p < 0.05, Figure 10). The increase in insulin in group B (6 to 7 mU/L; p = 0.06) and C (6 to 9 mU/L; p = 0.02) only reached significance for group C (Figure 7). Group B was the only group that had a postoperative increase in P-glucose (3.9 to 4.3 mmol/L; p < 0.05, Figure 8). These changes were not found in group D, in which insulin, glucose, HOMA and IGFBP-1 did not change significantly. IGF-1, however, decreased in both group B (139 to101 μg/L; p < 0.05) and group D (153 to132 μg/L; p < 0.05) the first postoperative day (Figure 6).

Amino acid infusion given to fasted volunteers did not affect any of the variables studied, neither on the first postoperative day nor during the study period.

## Discussion

Our study shows that an amino acid infusion during surgery attenuates the surgery-induced decline in IGF-1. This effect was most pronounced in the group who received amino acids throughout anesthesia. A decrease in IGF-1 during surgery is a general phenomenon described after both abdominal, heart and hip surgery [19,34-36] during different types of anesthesia. In the present study surgery was performed during the same type of anesthesia so the influence of anesthetic agents would not obscure the interpretation of the intervention.

The preserved IGF-1 levels found in the present study will most probably be a clinically important finding since reduced levels of IGF-1 are linked with postoperative catabolism. Protracted postoperative catabolism is a risk factor for complications and prolonged hospitalization. Administration of high doses of GH perioperatively has shown that retained IGF-1 levels are associated with improved nitrogen balance [37,38]. However, GH-treatment perioperatively is unsuitable since GH-treated critically ill patients, in two large randomized studies, had doubled mortality compared to their controls [39]. The mechanism behind this unexpected dramatic adverse effect by rhGH-treatment is still unknown but supposed to be caused by GHs immune modulating effect or GHs secondary negative effect on glucose homeostasis [40]. IGF-1 is not suspected and in a review IGF-1 was demonstrated to be safe [41]. Actually, maintained or restored IGF-1 levels have a potential to confer long term health benefits. Retained concentration of IGF-1 reduces the risk of diabetes, sarcopenia and cognitive attenuation [42,41] and in elderly also the risk of ischemic heart diseases [43]. Thus, to maintain IGF-1 after surgery in a cost-effective and safe manner may be beneficial.

Preserved levels of IGF-1 after surgery has previously otherwise only been reported when extremely high doses of insulin were given together with glucose and potassium (GIK) during heart surgery [35]. The present study shows that it actually also is possible to influence the postoperative decrease in IGF-1 with a balanced amino acid mixture supply.

The protracted "diabetes of injury" usually seen after surgery existed in the Ringer-group but was attenuated in the amino acid groups. In particular, amino acid infusion before and during surgery totally abolished the increase in insulin, and diminished the increase in IGFBP-1 and HOMA seen in the other operated groups on the first postoperative day.

Improved glucose control during cardiac surgical procedures decreases the risk for postoperative complications [44]. Furthermore, tight blood glucose control, with intensive insulin therapy, dramatically reduces mortality and morbidity in critically ill patients [30]. These findings indicate that our results, that amino acids infusion improves glucose homeostasis, might be clinically important.

There are some possible explanations why amino acid infusion might attenuate the surgery induced protracted insulin resistance. First, the preserved levels of IGF-1 in the amino acid groups might contribute since increased levels of IGF-1 is shown to reduce insulin resistance [8,9]. However, Skjaerbaek et al. found unchanged levels of free IGF-1 despite a significant decrease of total IGF-1 after surgery[45]. They suggest that the increased protelytic activity preserves the level of free IGF-1. Free IGF-1 was not measured in the present study so the preserved levels of IGF-1 may not completely explain the improved glucose homeostasis in the amino acid groups. Hence, other mechanisms had to be considered.

The balanced amino acid mixture used in the study contains L-Arginine, a precursor for NO. L-arginine acutely initiates the secretion of insulin, glucagon and GH. Both intravenous infusion [46], and long-term oral administration in diabetic patients [47] of L-arginine have been demonstrated to improve insulin sensitivity. The dose of L-arginine, given in our study, is compatible with the low-dose used by Wascher et al[46]. Hence, this effect of L-arginine might be of importance.

The greatest effect of a perioperative amino acid infusion on the short-acting IGFBP-1 and insulin was seen at the first day after surgery. On the day after surgery, the group receiving amino acid infusion before and during the first part of surgery (D) had lower levels of insulin than the patients receiving amino acids only during surgery (C). The most probable reason for this is the different regimes of amino acid administration. An infusion of amino acids in rats caused a decrease in IGFBP-1 without any changes in insulin [48]. A decrease in IGFBP-1 with unchanged insulin levels is known to reflect an improved hepatic insulin sensitivity [49]. Furthermore, the free glucose lowering fraction of IGF-1 depends on changes in IGFBP-1, the only binding protein demonstrated to have acute metabolic effects [50]. In the amino acid-treated rats, a higher concentration of total IGF-1 was detected [48] in agreement with our findings. Our results suggest that preoperative amino acid infusion has a pharmacological effect and preserves IGFBP-1 postoperatively in traumatized individuals. Consequently, the timing of the amino acids infusion might be of importance for the metabolic response.

Our patient groups were unfortunately not perfectly matched at baseline. The lower levels of IGFBP-1 and a tendency to greater body weight in group C, may suggest an alternative explanation of the difference in insulin pattern between groups C and D. Individuals that have low IGFBP-1 before surgery (patients C_1_, C_3_, C_6_) have also high insulin levels postoperatively, figures 1, 2, 3, 4, 5. From earlier studies it is known that obesity, low levels of IGFBP-1 and an increased IGF-1:IGFBP-1 ratio are factors associated with insulin resistance in men and women [49,51]. This possibly altered basal metabolism in some of the individuals in group C might be an additional reason for the different metabolic pattern between the two treatment groups.

In the Ringer-group we found a pathological metabolic pattern after operation, with depressed IGF-1 and protracted increased levels of glucose and insulin. In both treatment groups, amino acid infusion attenuated the surgery related decline in IGF-1, and improved glucose homeostasis. However, in none of the amino acid groups the amino acid regime were optimized. The optimal dose and timing of perioperative amino acid therapy need to be clarified in larger studies. The amino acid infusion could probably start well ahead of anaesthesia and continue during surgery. At least 60 minutes prior to anaesthesia seems to be appropriate. A possible advantage of a continued infusion after operation has also to be investigated. The exact mechanism behind this favourable effect has also to be elucidated in further studies.

## Conclusion

Our study shows that amino acid infusion during surgery reduces the decrease in IGF-1. It also indicates that amino acid therapy before and during surgery diminishes "diabetes of injury". Larger studies are required to find out doses, timing and mechanisms behind the favourable effects of amino acid therapy during surgery.

Our findings might change the perioperative infusion therapy in the future.

## Abbreviations

IGF-1 = Insulin-like growth factor-1

GH = Growth hormone

IGFBP-1 = Insulin-like growth factor binding protein-1

HOMA = Homeostasis model assessment

RIA = Radioimmuno assay

## Competing interests

The author(s) declare that they have no competing interests.

## Authors' contributions

MW and ES conceived the study; MW drafted the manuscript; ES was responsible for the clinical data collection and helped to draft the manuscript; SE acted as a statistical consultant and made the figures; KB was responsible for the laboratory analysis, helped to draft the manuscript and rose the funding that made the study possible. All authors have substantially contributed to the manuscript and approved the final manuscript.
